# Time for international action on treating testosterone deficiency syndrome

**DOI:** 10.1080/13685530802699067

**Published:** 2009-03-26

**Authors:** Malcolm Carruthers

**Affiliations:** Centre for Men's Health, London, UK

**Keywords:** Expert, opinion, testosterone deficiency syndrome, TDS, treatment, prevalence, education

## Abstract

**Aim:**

Testosterone deficiency is having an increasing impact on men's health because of global aging, higher levels of obesity, diabetes and metabolic syndrome and adverse environmental factors such as stress xenoestrogens and anti-androgens. The question addressed is to what extent the large body of evidence on the benefits and safety of testosterone therapy is applied in clinical practice.

**Methods:**

Demographic data for men over the age of 50 from different regions of the world have been compared with the number of men in that age group estimated from sales figures to be receiving testosterone treatment.

**Results:**

On the basis of estimate that 20% of men over 50 in the general population of each region could be expected to have testosterone deficiency symptoms, on average only these men (0.69%) in most European countries were receiving treatment. Proportion was higher in the UK (1.00%) and Germany (1.89%), but lower in France (0.49%), Italy (0.51%) and Russia (0.54%). Interestingly, Australia had higher figures (1.64%), in spite of tight state control measures on androgen use. The USA has the highest treatment rate (7.96%) and this is increasing rapidly.

If the basis for the diagnosis was the more conventional combination of symptoms plus biochemical evidence of low total and free testosterone levels, androgen deficiency would be expected in at least 5% of men over 50, and percentage treatment rates therefore four times higher. However, even on that basis, only in the USA do these exceed 10%.

**Conclusions:**

International action is urgently needed to raise awareness in the medical profession in the various countries of these remarkably low levels of testosterone treatment. Improvement in this requires education and motivation of doctors and those regulating the healthcare systems. A public awareness campaign is needed to educate men about the symptoms of testosterone deficiency and its impact on their health.

## Introduction

*Global Warming – ‘February 2nd 2007 will be remembered as the day when global thinking about climate change moved from debate to action’ (Achim Steiner Executive Director UN Environment Program)*.

This Millenium has seen rapidly growing awareness of the important role that testosterone plays in maintaining men's health and quality of later life. Testosterone deficiency is now recognised as a very real and increasing problem for several reasons.

Global aging is occurring rapidly, particularly in both developed and developing countries, but an increased life expectancy is not unfortunately being accompanied by greater health expectancy – the gap is widening. With age, testosterone levels fall, and there is greater resistance to its action, partly because of rising Sex Hormone Binding Globulin (SHBG) levels causing a more marked drop in bioavailable and free testosterone (FT) [1]. This reduction in testosterone is made worse by illness and the drugs used to treat it, especially in diabetes and metabolic syndrome, both of which are increasingly exponentially. There is some recent evidence that testosterone levels are falling generally in men, both as seen in cohorts of the population in Denmark [2] and in the USA [3], and this is attributed to both lifestyle and environmental factors. Included in the environmental causes are likely to be the increasing effects of xenoestrogens and anti-androgens, which in younger men appear also to be having a potentially serious effect on reproductive health [4].

Not only is the quality of a man's life greatly reduced by the characteristic symptoms of testosterone deficiency, which include loss of energy and libido, erectile dysfunction, irritability, joint pains and stiffness, memory impairment, irritability and depression, but there is well documented associated morbidity and mortality in relation to a wide range of serious conditions. There are many epidemiological, theoretical and therapeutic studies linking low androgen levels in men with cardiovascular disease [5,6], metabolic syndrome [7,8], diabetes [9,10], osteoporosis [11,12], frailty [13,14] and Alzheimer's disease [15–17]. As might be concluded from these now well recognised associations, life expectancy is also decreased by androgen deficiency (AD) [18,19], and may partially explain the greater longevity of women. This is emphasised by Wu et al. in the European Male Aging Study [20], which shows the complex multiple alterations in the hypothalamo – pituitary – testicular axis function associated with progressive age-related testicular impairment as well as specific risk factors, and emphasises the importance of maintaining testosterone levels in aging men.

Like global warming, awareness of the effects of testosterone deficiency is also increasing as the general public surf the oceans of information available on the internet, but still the condition remains largely unrecognised and untreated. The symptoms of ‘Testosterone Deficiency Syndrome’ (TDS) as it has come to be called in preference to andropause or late-onset hypogonadism, were considered relatively rare in the 1940's when it was recognised as the ‘male climacteric’, but are now frequently seen in middle-aged and older men.

## Prevalence of TDS

The prevalence of TDS in different countries is a matter of intense academic debate. Estimates of its frequency in the general community vary as widely as between 0.5% [21] and on a symptomatic basis in men over 50, nearly 50% [3], a 100-fold difference.

The answer depends on the population of men studied, their age, and whether symptoms alone are considered diagnostic or additional laboratory criteria such as testosterone level are required. These polar views both need to be considered, because the biochemical criteria can be questioned on the grounds of validity of androgen assays [22], and their significance in the light of the concept of androgen resistance, akin to that seen with insulin in Type 2 diabetes mellitus [1].

Large-scale epidemiological studies such as the Boston Area Community Health (BACH) Survey [3] have shown that while 37.7% of men over the age of 50 reported symptoms of AD, only 15% had low total testosterone (TT), 9.9% low FT and 8.4% both. This left 15% with low levels of TT or FT, and no symptoms. The very poor overlap of symptoms and biochemistry seen in most clearly in Venn diagrams resulting from such studies raises the question of who should be given access to a therapeutic trial of testosterone, the patients with symptoms, even if they lack a biochemical passport or only those with both?

Using the best validated and most widely studied questionnaire, the Aging Male Symptoms (AMS) scale [23,24], Heinemann found a prevalence of moderate and severe symptoms of testosterone deficiency in around 20% for European men over 50 [25] from an aggregate of studies in Germany, UK, France, Spain, Portugal, Italy and Sweden. This international comparison, using the questionnaire meticulously translated into the various languages [26] showed closely similar correlations between the total scores and sub-scales, both in ‘community populations’ in all countries studied, and in patients in urology clinics in Germany and Austria [27].

Like all questionnaires designed to detect symptoms of AD, the AMS scale shows little or no correlation with either TT or other endocrine measures such as calculated free testosterone (CFT) [28]. Importantly, it is however an efficient means of predicting and measuring clinical response to symptoms of testosterone deficiency, which could be taken as a true measure of its clinical validity. Of 1670 urology clinic patients treated with Testogel for 3 months, 73% had moderate (35%) or severe (38%) symptoms before, and only 22% afterwards, (20% moderate and 2% severe), a much more normal picture [29]. This improvement was not related to age, BMI or testosterone levels. Similar results were obtained in a study of patients in another German urology clinic given testosterone enanthate injections [28], showing the symptomatic response is not dependant on testosterone preparation providing the dosage is adequate.

All self-reported questionnaires have good sensitivity, but low specificity in relation to androgen values. The St. Louis Androgen Deficiency in the Aging Male (ADAM) questionnaire [30] has been extensively used, but some of the claimed correlation with testosterone levels are likely to be age-related [31]. Although the ANDROTEST questionnaire gave higher specificity than the ADAM in relation to both TT and FT [32], clinical experience with it is limited as it has only been used by its originating group in the 2 years since it was published.

Because it is fully recognised that the current consensus view of the major societies in the field require biochemical confirmation of the diagnosis, figures for those with additional biochemical evidence, as well as those with symptoms alone, will be given later in this article.

## Prevalence of testosterone treatment

It is of importance to compare the number of men over 50 in various countries, for which accurate figures are available from official demographic surveys such as Eurostats, US Census Bureau, Health Statistics from the Americas 2006 (Pan American Health Organisation) and Australian Bureau of Statistics, with testosterone usage in those regions (Tables I and II).

**Table I:** Numbers of men in millions at risk of TDS in various age categories, estimates being made around 2006.

Region	Age 50–64	Age 65–79	Age 80+
Whole EU	42.53	29.61	9.63
UK	4.99	3.26	1.23
Ireland	0.33	0.17	0.04
Spain	3.44	3.25	1.23
Italy	5.23	4.17	1.46
France	5.32	3.45	1.36
Germany	6.48	5.21	1.58
America	25.14	11.76	3.82
Russia	10.60	4.90	1.50
Australia	1.72	0.94	0.27

**Table II:** Number of men over 50, and those expected to be androgen deficient on the basis either of symptoms alone (20%), or with symptoms and low TT and CFT (5%), compared with total number being treated in each region. Estimates are made for around 2006.

Region	Men aged >50 (Millions)	TDS symptoms (Millions)	Total number treated	Symptoms treated (%)	Sympt. + biochem. (Millions)	Symptoms + biochem treated (%)
Whole EU	81.77	16.35	113,000	0.69	4.09	2.76
UK	9.48	1.90	19,000	1.00	0.47	4.00
Ireland	0.54	0.11	1,000	1.10	0.27	4.40
Spain	7.92	1.58	12,000	0.76	0.40	3.04
Italy	10.86	2.17	11,000	0.51	0.54	2.03
France	10.13	2.03	10,000	0.49	0.51	1.97
Germany	13.27	2.65	50,000	1.89	0.66	7.55
America	40.72	8.14	648,000	7.96	2.04	31.84
Russia	17.00	3.40	18,400	0.54	0.85	2.16
Australia	2.93	0.59	9,100	1.54	0.15	6.17

The number of men on testosterone treatment is difficult to estimate accurately because moving annual turnover figures from different governmental and commercial agencies often give only the monetary value and number of packs of testosterone sold. Each pack may provide a variable duration of treatment, depending on the preparation and dosage.

However, assuming that one ampoule of mixed testosterone esters or testosterone enanthate last on average 3 weeks, this means that 18 single dose packs are dispensed for each patient yearly. Similarly, some oral preparations such as testosterone undecanoate are dispensed in packs lasting for either 2 weeks or a month, as are testosterone gels and patches. Long-acting testosterone undecanoate injections last 3 months, and testosterone implants last about 1 month per 200 mg pellet, assuming six pellets are implanted every 6 months. Given figures for the number of packs of each preparation prescribed per year, as are available for the UK, Russia and Australia, it is possible to obtain a fair approximation for the number of patients receiving testosterone treatment in each country. These figures are only estimates, and may well have to be revised up or down as more accurate data becomes available from international studies.

Unfortunately, there is also no indication of the proportion of treated patients who are receiving treatment for conditions other than adult onset testosterone deficiency e.g. Klinefelter's Syndrome estimated at 0.2%, but undiagnosed in many cases [33], undescended testis (0.2%) [34], which also remains undiagnosed or untreated in over 50% of cases. Though excluded where possible in these analyses, prescriptions for women have been estimated at 0.05% (IMS figures), and are probably increasing especially in the US where surprisingly, as the toxic methyl testosterone, they have long been a component of hormone replacement regimes.

Table II shows the number of men over 50 years who are estimated to have symptoms of testosterone deficiency compared with the number of men receiving treatment, based on one of two assumptions. Firstly, if the diagnosis deficiency is made on presence of symptoms alone, for example a moderate to severe score on the AMS scale, based on the existing figures it would be reasonable to assume a prevalence of 20% in men over the age of 50.

Alternatively, if in spite of the low degree of overlap, both symptomatic and biochemical evidence in the form of low TT and FT is required to confirm the diagnosis, only about 5% could be expected to have AD [3], which quadruples the percentage of cases treated in each region.

With either scenario, the number of cases treated includes half the cases of Klinefelter's Syndrome, i.e. 0.1%, and half of the undescended testes, i.e. 0.1%. The estimates are based on 2006–2007 figures from Intercontinental Medical Statistics. (IMS), Bayer-Schering Russia and Medicare Australia.

## USA

In a few countries, notably the USA which now represents about 90% of the market for testosterone products, the treatment is becoming more widely used, but in most usage is static and minimal, as pharmaceutical companies trying to introduce new preparations elsewhere have found to their cost. The rapid expansion in the USA is largely because of rapidly growing public awareness and enthusiasm for testosterone treatment fostered by the internet, together with safer and more convenient products, as well as easier access though private physicians rather than in a state-funded system.

There the market for testosterone therapies has increased from US $49 million to almost $400 million between 1997 and 2003, with the majority of prescribing being for men 40 years and older [35]. It has been reported that the average annual increase in prescriptions and patients receiving testosterone treatment had reached 29% by 2002 [36], so that total sales were likely to have gone over the 1 Billion $US mark by 2007.

However, the same detailed and authoritative ‘Testosterone and Aging’ report from the US Board of Health Sciences Policy in 2004 [36] using US census data estimated that in 2002, 1.14% of 46–65 year old males were using testosterone products (58% of total retail patient count), compared with 0.56% of men over 65 (13% of total retail patient count), and that this was increasing by about 20% annually in both groups. Linear extrapolation, which again may well be an underestimate especially in view of the rapid increase in illicit Internet sales, gives a frequency of usage of 31.8% of biochemically confirmed cases in 2006.

Allowing for 20% annual increase in usage, and higher frequency of AD in older age groups, these figures are supported by a recent study in the BACH Survey, showing that of the 5.5% of men in the age range 30–79 years in 2002 who fulfilled their criteria of AD, i.e. had symptoms of AD together with low total and FT, only 12.2% were receiving testosterone treatment [37]. The authors conclude that ‘a large majority of men in our groups with AD were not receiving treatment despite adequate access to care. The reasons for this are unknown but could be because of unrecognised AD or unwillingness to prescribe testosterone therapy’. These are important themes are taken up later in this article.

## Europe

By contrast, according to figures for Europe and the UK provided by IMS and British Pharmaceutical Index (BPI Data), compared with demographic figures from Eurostats, testosterone usage in Europe is generally much lower, averaging 1% or less of men with symptoms, except in Germany where it is better recognised and more accepted. This is in spite of recent statements by European authorities that ‘Measurements recorded to date suggest that ∼12% of men aged 50–59 years, 19% of men aged 60–69 years, 28% of men aged 70–79 and even 49% of men over 80 suffer from hypogonadism’ [38]. Also, the market in Europe is virtually static, increasing by only 0.2% between 2007 and 2008.

## Russia

In Russia, there is a rapid reduction in life expectancy for men, which has now fallen to about 58 years because of factors such as psychosocial stress and the ravages of alcohol. This results in a rapid fall off in the number of men over the age of 50, and a high prevalence of TDS in this group due to the same factors.

Analysis of the testosterone market in Russia shows doubling of the money spent on all testosterone preparations between 2001 and 2007. Unfortunately, this obscures an estimated halving of the number of men receiving any testosterone treatment, largely due to greatly reduced prescribing of the low cost but toxic methyl testosterone which was given in over 75% of cases at the beginning of the period, and still over 50% in 2006. This emphasises the need for economic but safe products such as creams and gels which can be applied to the scrotal skin, the site of maximal transdermal absorption and hence economic and convenient use (Table III).

**Table III:** Absorption, theoretical and practical advantages, together with costs, of each preparation and their application at NHS prices (£), giving a monthly total treatment cost, excluding medical costs of diagnosis and monitoring treatment. Theoretical and practical aspects of each treatment are rated from *poor, **moderate, ***good, to ****excellent.

Testosterone preparation	% Abs.	Theory	Practice	Cost	Appl.	Total
Testosterone pellet implant	100	*	**	14	50	64
Injected T-esters (Sustenon)	100	*	*	5	20	25
Injected T-undecanoate (Nebido)	100	**	***	25	5	30
Oral T-undecanoate (Testocaps)	10	**	**	36	0	36
T-Gel (Testogel, Tostran, Testim)	15	***	***	33	0	33
Scrotal T (Andromen, Tostran)	70	****	****	13	0	13

## Australia

A rising trend in testosterone prescribing which started in the mid-1990s was suppressed by two successive rafts of legislation [39]. Combining Australian Bureau of Statistics figures for 2006 with those of Medicare Australia for Pharmaceutical Benefit prescriptions for 2006, it is estimated that ∼1.54% of the men over 50 likely to have TDS symptoms were receiving testosterone treatment, and of those with biochemically proven AD, just over 6% were being treated.

## Causes of failure to treat androgen deficiency

### The diagnosis of androgen deficiency is missed because of over-emphasis on laboratory data at the expense of clinical data, especially symptomatology

Led by epidemiologists, there has been a rush to devalue and then dismiss the well-established clinical symptoms of testosterone deficiency. This is on the grounds that there is very poor correlation between these and laboratory data, and that any links between the two, especially when starting with non-specific questionnaires such as the ADAM, are age-related. Typical of this was a study by Spetz et al. [40] in an elderly Swedish population where as with other studies, only a weak association was found between symptoms in the ADAM questionnaire and blood testosterone concentrations. On this basis, Mckinlay in an editorial in the same issue of the Menopause Journal concluded that the idea of a ‘hormonal syndrome’ should be finally declared dead and given a ‘decent burial’ [41].

However, also in 2007, the same group published the previously quoted article on ‘The prevalence of symptomatic AD in men’ [3], in which they posed the question ‘What are the clinical risks of missing these asymptomatic men with low testosterone levels, if any?’ Given that in the large majority of cases, regardless of age and androgen levels, the symptoms as measured by the carefully designed, quantifiable and non-aged related AMS scale can be reversed [29], perhaps the question could be viewed from another angle. – What are the clinical risks of missing symptomatic men with ‘normal’ testosterone levels?

It is suggested that given characteristic symptomatology, particularly in the presence of medical conditions known to be associated with TDS, setting arbitrary cut-off points for TT or CFT above which AD can be definitely excluded is both scientifically and clinically unjustified. This contention is supported by the existing evidence on the inconsistencies in sampling procedures, laboratory analyses and uncertainties about the interpretation and significance of so-called ‘normal ranges’ of androgens [22].

These problems surrounding the laboratory-based diagnosis of TDS are compounded by the evidence that there can be both insufficient production, and variable degrees of resistance to the action of androgens operating at several levels in the body simultaneously. These factors become progressively worse with aging, adverse life-style, other disease processes, and a wide range of medications [1].

### Unfounded concerns about the side-effects of treatment, especially in relation to prostate cancer

In a world-wide study of the attitude of physicians to treating testosterone deficiency, apart from uncertainties over diagnosis, by far the commonest barrier in every country was fear of inducing prostate cancer (pCA) (60%) [42]. This was combined with unfounded concerns over the possible adverse effect on benign enlargement of the prostate [43], and also lack of awareness of the important role of T in erectile dysfunction [44,45]. It is suggested that physicians need to be updated on all these points.

The particular, the current state of knowledge on T and pCA is best summarised by the statement in the article that ‘current literature does not provide any evidence of a cause – effect relationship between endogenous T or T treatment and pCA development’. Further, the concerns about any such link been declared to be ‘A modern myth’ by the American urologist Morgentaler who goes as far as to state ‘that there is not now – nor has there ever been – a scientific basis for the belief that T causes pCA to grow’ [46].

The overly cautious attitude of many physicians is to suggest awaiting the out-come of large-scale randomised placebo controlled trials, even though these are unlikely to be funded or completed in the lifetimes of most men currently needing treatment. Further delay on these grounds also seems unjustified in the light of the many studies carried out before all recently licensed products were released, as well as extensive post marketing surveillance, together with 70 years clinical experience of safe and effective testosterone treatment.

### Lack of awareness of the many serious general medical conditions in which androgen deficiency plays a major role [47]

As referenced above, AD has been linked epidemiologically and clinically to many of the commonest, most severely debilitating and distressing afflictions of aging men, including cardiovascular disease, metabolic syndrome, diabetes, osteoporosis and Alzheimer's disease. As well as its overall health benefits, sexual health is improved by increased libido, and testosterone is increasingly recognised as a adjunct to PDE5 inhibitors in treating erectile dysfunction [45]. Finally, testosterone treatment can reduce the distressing and expensive period of frailty and dependency at the of end life.

The argument that many of the symptoms will remit if left untreated [48] does not take into account the damage done by the above conditions to the man's general health and quality of life while waiting for the spontaneous which might occur 10–15 years later in 50% of cases.

### Lack of training in safe and effective testosterone treatment

Guidelines for this have now been provided by the International Society for Andrology, the International Society for the Study of the Aging Male and European Association of Andrology [43], as well as the Endocrine Society in America [49], and are remarkably uniform in their safety recommendations. An on-line training course based on these guidelines has recently been established by the Society for the Study of AD, and for the first time makes the theoretical and practical information needed to diagnose and treat AD available to physicians anywhere in the world (www.andropause.org.uk).

### Concerns about the cost of treatment and failure to appreciate the true long-term costs of not treating this condition

There is a lack of awareness of recent advances in safe and economic forms of androgen treatment, and the continuing availability of toxic products such as methyl testosterone e.g. in France and Russia [50]. This has tended to over-shadow the economic as well as the medical burden of TDS in terms of both morbidity and mortality [51].

The costs of testosterone treatment have not been studied in any detail, but need to be part of any convincing cost/benefit analysis. Table III compares the features and monthly costs of the most commonly used testosterone preparations in the UK at NHS prices. Absorption varies widely from the total utilisation of implanted and injected forms, to the poor and variable absorption of the oral preparations. Theoretical advantages of each preparation have been rated according to the degree to which the preparation restores a steady and natural physiological endocrine pattern, avoiding supraphysiological peaks of testosterone, and suppression of LH and FSH which can cause distressing testicular shrinkage. Practical considerations include the need for 6 monthly implant appointments, twice monthly injections by a nurse in the case of the injected T-esters, or twice daily dosage with oral treatments, and the patient's preference for the various forms of treatment. Also, as in the case of pellet implants and injected T-esters, the costs of the preparation may be exceeded by those of its application by scarce medical and nursing staff.

In well developed health-care systems such as that in the USA, there is emphasis on the concept of ‘preventive care’ in limiting spiraling costs in this major segment of the economy, though there is considerable debate over which clinical interventions actually save money [52]. These authors raise the key point that ‘Findings that some cost-saving or highly efficient measures are underused would indicate that current practice is inconsistent with the efficient delivery of health care’. Testosterone treatment can provide short and long-term improvement in the quality of life, and be of benefit in many serious and debilitating conditions, when carefully targeted it could be considered a strong candidate for providing cost-effective preventive medical care.

## Conclusions

International action is urgently needed in making these problems known to the medical profession in each country, and overcoming the obstacles to treatment by educating and motivating them and those regulating the healthcare systems, as well as encouraging the public to be aware of symptoms of TDS and its importance.

The proposed sequence of events is firstly that primary care physicians should be made aware that AD is important, frequently occurring, easily diagnosed by questionnaires such as the AMS, and safe, simple and economic to treat. Without this initial stage, many patients with diagnostic symptoms will continue to go unrecognised and untreated.

Secondly, specialists such as urologists, especially those treating erectile dysfunction, andrologists, endocrinologists seeing patients with diabetes and metabolic syndrome, cardiologists, psychiatrists and gerontologists should all be more aware of the need to recognise and treat the condition.

Last but not least, by un-sensational but authoritative TV programs and articles in the popular press, we can educate the general public, increasing their awareness of the symptoms and effects of testosterone deficiency, and the efficacy of treatment. The economic case that it could be an important part of preventive medical care in aging men with testosterone deficiency needs to be developed and publicised.

Like climate change, it could be said that the time has come when global thinking on the need to diagnose and treat testosterone deficiency should move from debate to action.
